# Natural history and treatment of asymptomatic unruptured carotid cavernous aneurysms: a systematic review and meta-analysis

**DOI:** 10.1007/s10072-025-08584-y

**Published:** 2025-12-20

**Authors:** Gianluca Trevisi, Giovanni Pennisi, Samuele Santi, Nicola Montemurro, Matteo Palermo, Carmelo Lucio Sturiale

**Affiliations:** 1 Department of Neurosciences, Imaging and Clinical Sciences, G. D’Annunzio University, Chieti-Pescara, Italy; 2 Department of Neurosurgery, Fondazione Policlinico Universitario A. Gemelli IRCCS, Università Cattolica del Sacro Cuore, Rome, Italy; 3 Department of Neurosurgery, Azienda Ospedaliero Universitaria Pisana (AOUP), Pisa, Italy; 4 Institute of Neurosurgery, Università Cattolica del Sacro Cuore, Rome, 8-00168 Italy

**Keywords:** Carotid cavernous aneurysms, Endovascular treatment, Intracranial aneurysms, Natural history, Neuro-ophthalmic symptoms

## Abstract

**Background:**

The management of asymptomatic carotid cavernous aneurysms (CCAs) is debated. While their rupture risk is low, the potential for growth and symptom development creates a clinical dilemma, particularly with the evolution of advanced endovascular treatments. This study aims to synthesize the evidence on the natural history of asymptomatic CCAs and the outcomes of intervention.

**Methods:**

A systematic review of PubMed and Scopus was conducted per PRISMA guidelines. We included retrospective studies reporting on the management of asymptomatic CCAs with five or more patients. A proportional meta-analysis was performed to calculate pooled outcomes for conservative and active management cohorts separately, as the observational nature of the included studies precluded direct comparative analysis.

**Results:**

Eleven studies met the inclusion criteria, encompassing 557 patients. Conservative management was the initial strategy in 75.0% of cases (n=418), while 25.0% (n=139) underwent active treatment. In the conservatively managed cohort, 8.1% of patients developed new neuro-ophthalmic symptoms. Radiologically, 91.5% of followed aneurysms remained stable. Aneurysm size was the strongest predictor of progression, with large aneurysms (≥12 mm) showing a 19.2% annual growth risk. Active treatment achieved a pooled aneurysm occlusion rate of 85.7% but was associated with complications, including a 2.9% rate of ischemic events.

**Conclusion:**

The natural history of asymptomatic CCAs is largely benign, but progression is strongly linked to aneurysm size. Our findings support a risk-stratified approach: conservative management with serial imaging for small (<12 mm) aneurysms and consideration of intervention for larger or growing lesions.

## Introduction

Carotid cavernous aneurysms (CCAs) are a unique neurovascular pathology, accounting for 2–9% of all intracranial aneurysms [1]. Their location within the cavernous sinus, an extradural space, dictates a clinical profile distinct from their intradural counterparts [2]. The primary risk is not life-threatening subarachnoid hemorrhage but rather neurological morbidity from mass effect on adjacent cranial nerves, leading to neuro-ophthalmic deficits such as diplopia and ocular pain, which are the main indications for treatment [3].

The increasing incidental discovery of asymptomatic CCAs presents a significant therapeutic dilemma [4]. While a recent comprehensive meta-analysis has robustly clarified the natural history of conservatively managed CCAs by calculating precise annual risks for growth and symptom progression [5], there remains a need to synthesize the outcomes of active intervention alongside these observational data. This systematic review aims to address this gap by analyzing pooled data from both conservatively and actively managed cohorts to fully inform the clinical decision-making process for the asymptomatic patient.

## Methods

This systematic review was performed and reported according to the PRISMA (Preferred Reporting Items for Systematic Reviews and Meta-Analyses) 2020 guidelines [6].

### Search strategy and study selection

Two authors (GT and GP) performed a comprehensive search of the PubMed/MEDLINE and Scopus databases to identify relevant studies. The search terms used were: “(intracranial OR brain OR cerebral) AND aneurysm AND (paraclinoid OR cavernous OR intracavernous OR syphon OR ‘internal carotid artery’ OR ‘carotid artery’)”. The search was updated to December 31st, 2024, and a forward search of reference lists from retrieved articles was also performed to increase search power.

The inclusion criteria were as follows: (1) peer-reviewed studies published in English reporting on the management of asymptomatic internal carotid artery aneurysms located in the cavernous sinus; (2) studies including more than five patients; and (3) studies containing quantitative data on outcomes. We excluded case reports, comments, letters, and review articles. Studies that did not provide explicit or separable data for asymptomatic CCAs versus symptomatic ones were also excluded. Two authors (CLS and GP) independently screened titles and abstracts, followed by a full-text review of potentially eligible articles to determine final eligibility. Disagreements were resolved by consensus.

## Data extraction and outcomes of interest

For each eligible study, two authors independently extracted the following data: study details (author, year, design); patient groups (total number of patients per treatment arm); treatment modalities; and outcomes. The primary outcomes of interest for this review were, for the conservative management group, the rates of aneurysm stability, aneurysm growth, and development of new neurological symptoms. For the active treatment group, the primary outcomes were the rate of aneurysm occlusion and the rate of procedure-related complications.

## Quality assessment

The Risk Of Bias In Non-randomized Studies - of Exposure (ROBINS-E) tool was used to assess the methodological quality. This tool was chosen because it is specifically designed for non-randomized, observational studies that examine the effect of an exposure (i.e., the presence of an aneurysm or a specific management strategy) on outcomes. This framework allows for a focused evaluation of biases inherent in such studies, including confounding, selection bias, and classification of exposure.

### Statistical analysis

Recognizing that the included studies are primarily single-arm, retrospective series without comparative cohorts, a direct statistical comparison between conservative and active treatment using metrics like relative risk is inappropriate and methodologically unsound. Therefore, we performed a proportional meta-analysis. This approach allowed us to calculate separate pooled estimates for key outcomes within each management arm. A random-effects model was chosen to account for the significant clinical and methodological heterogeneity expected across studies. Statistical heterogeneity was quantified using the I^2^ statistic. All statistical analyses were conducted using OpenMetaAnalyst software (Brown University, an R-based program funded by the AHRQ, Rockville, MD, USA).

## Results

### Study selection and patient characteristics

**Fig. 1:**
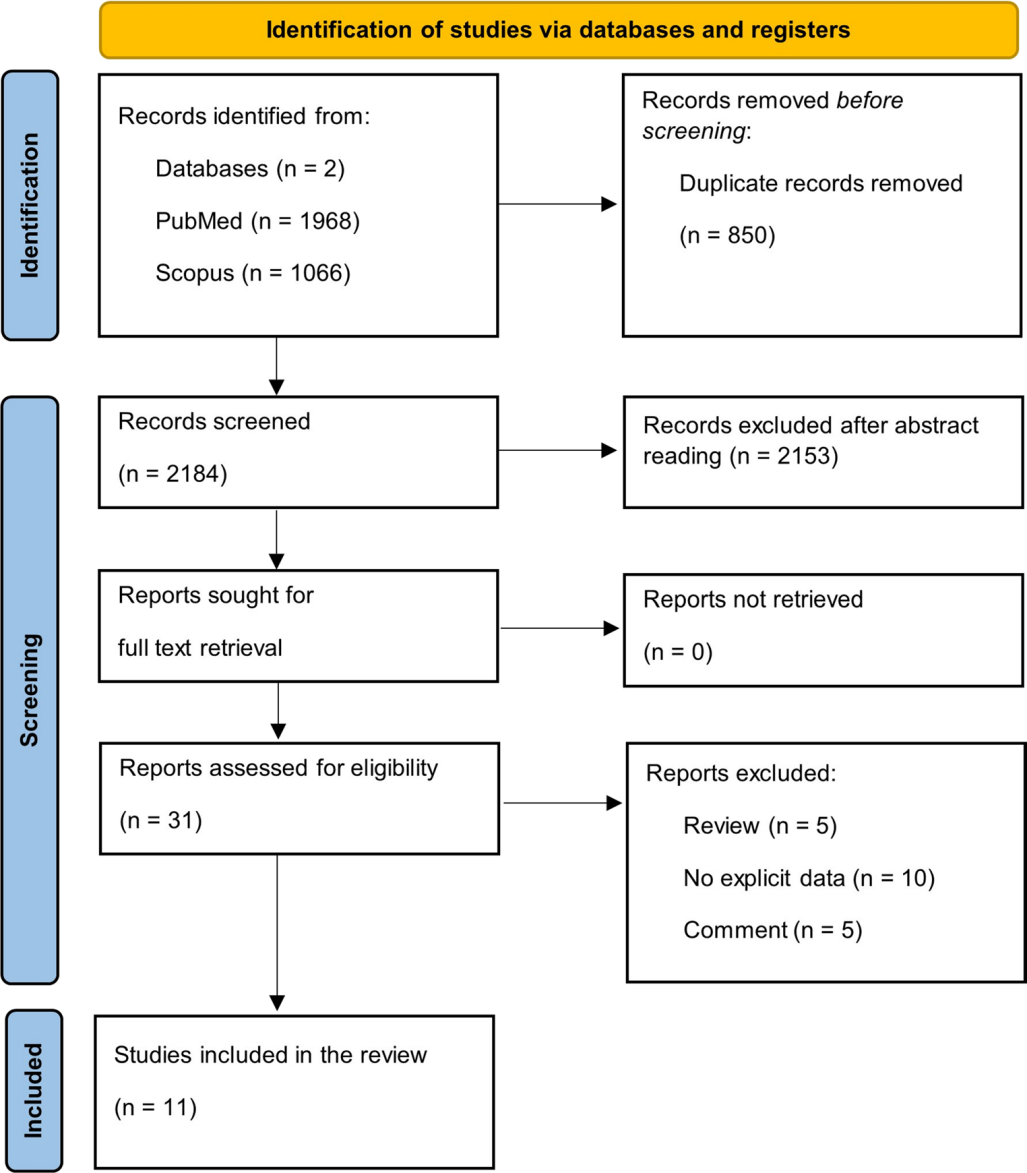
PRISMA diagram

A comprehensive search of PubMed and Scopus initially identified over 3,000 studies. After applying strict inclusion criteria—which involved excluding duplicates, case reports, non-English studies, and articles with incomplete data or fewer than five patients—a final cohort of 11 retrospective studies met the eligibility requirements for this review [7–17] (Fig. 1). A critical aspect of our methodology was the careful stratification of patients; to maintain methodological rigor, only data explicitly labeled by the original authors as pertaining to asymptomatic aneurysms were extracted.

This process yielded a total of 557 patients with asymptomatic CCAs across the 11 studies. Of these, 418 patients (75%) were managed conservatively, while 139 (25%) received active treatment. Analysis of the patient demographics revealed several key trends. A strong female predominance was evident across the literature, with multiple series reporting that up to 92% of patients were female [14, 15]. The majority of patients were diagnosed in their sixth decade of life, with mean ages consistently reported around 60 years [11, 15].

Regarding aneurysm characteristics, the incidence of concomitant intracranial aneurysms was high, reaching 54% in one report, with a significant proportion of CCAs discovered incidentally following the rupture of an aneurysm in another location [14]. Bilateral (mirror) CCAs were also frequently observed [15, 17]. Aneurysm size was a significant factor, with giant CCAs (> 25 mm) constituting over 30% of cases in one large series [14]. Another study specifically highlighted that these larger aneurysms had the highest risk of growth, with a calculated annual growth rate of 19.2% per patient-year [17]. The mean follow-up period across the studies was 6.3 years, though the duration varied widely from 6 months to as long as 46 years [12].

## Natural history and outcomes of conservative management

For the 418 patients managed conservatively, the clinical course was largely benign. Across the entire cohort, only 34 patients (8.1%) developed new neuro-ophthalmic symptoms, primarily visual disturbances and trigeminal pain [14, 15, 17]. Importantly, no cases of aneurysm rupture or carotid-cavernous fistula were reported in this group.

Detailed radiological follow-up was available for a subset of 200 patients, providing further insight into aneurysm behavior. Of these, a vast majority of aneurysms, 183 (91.5%), remained stable, while 10 (4.9%) exhibited growth and 6 (3.0%) underwent spontaneous thrombosis [10, 14, 15, 17].

**Fig. 2:**
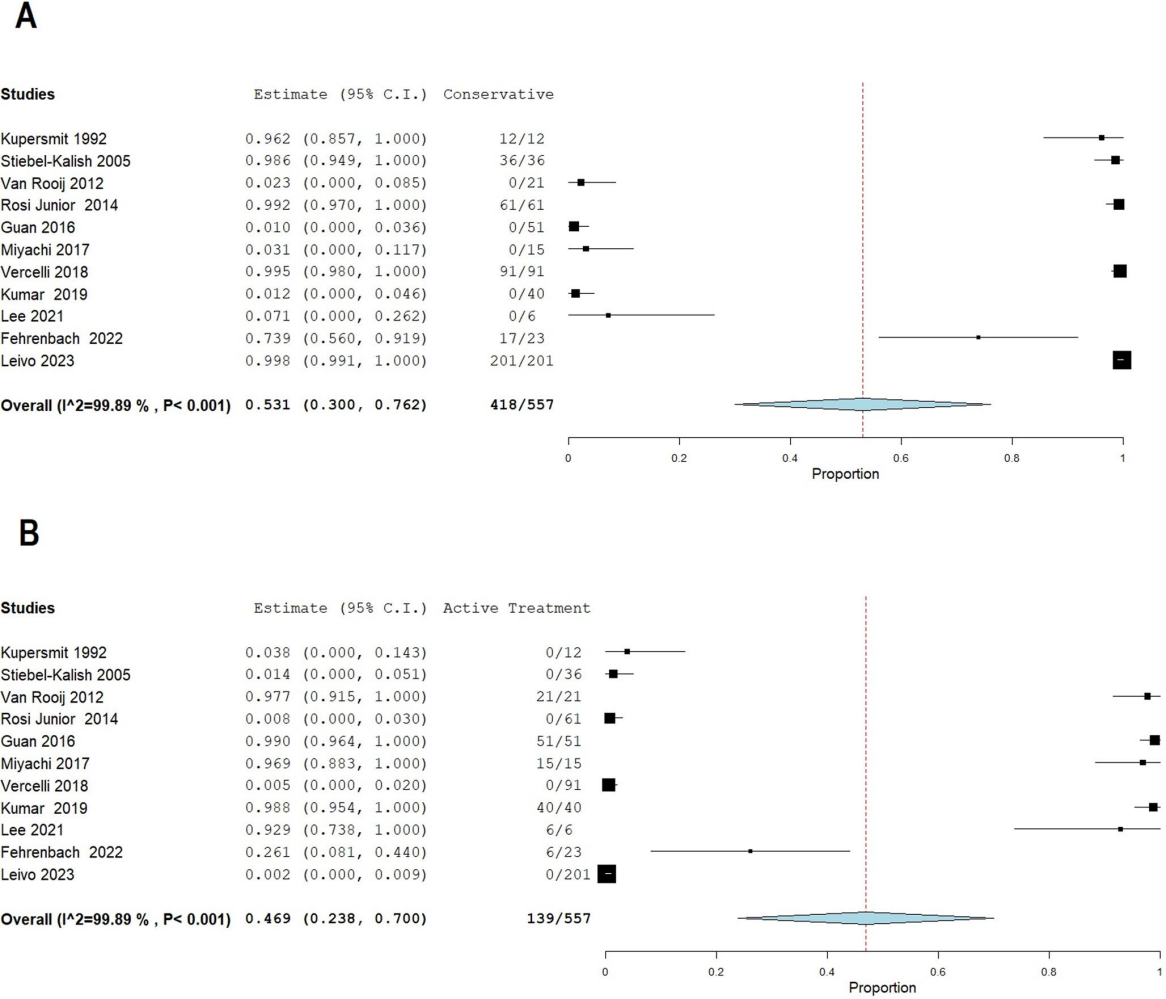
**A**. Prevalence of conservative approach; **B**. Prevalence of active treatment

Analysis of these outcomes revealed that aneurysm size was the most significant factor predicting progression. A study focusing specifically on growth risk found that large or giant aneurysms (≥ 12 mm) had a 19.2% annual risk of growth—a figure dramatically higher than the 1.2% risk for very small aneurysms (< 4 mm) [17]. This link between size and clinical events was further corroborated by reports showing that the mean aneurysm diameter in symptomatic groups was nearly double that of asymptomatic groups (15.3 mm vs. 8.1 mm) [8], and that the incidence of cranial nerve deficits increased with aneurysm size [12]. The pooled proportion of these outcomes is visually summarized in the Forrest plot (Fig. 2A).

## Outcomes of active intervention

The pooled proportion of actively treated patients is visually summarized in the Forrest plot (Fig. 2B).

Among the 139 treated patients, endovascular techniques were the most common modality. Interventions included endovascular coiling (60 patients), stenting (43 patients), combined coiling and stenting (12 patients), and flow diversion (12 patients). Surgical approaches, such as parent vessel occlusion or bypass, were used in a smaller number of cases.

Radiological outcomes were available for 124 of the treated patients. At follow-up, complete or near-complete aneurysm occlusion was achieved in 112 of these 124 cases, corresponding to a pooled occlusion rate of 85.7% (95% CI: 73.0–98.4%). A comparative study of different endovascular strategies noted that while Parent Artery Occlusion (PAO) resulted in immediate aneurysm disappearance, Flow Diversion (FD) also provided excellent final results with aneurysm shrinkage over time [13].

However, treatment is not without risk. One series reported major complications including two ischemic strokes following PAO (a rate of 16.7% in their cohort) and one iatrogenic aneurysm rupture during an FD procedure (a rate of 7.1% in their cohort) [11] (Table 1.).

**Table 1.** Summary of included studies

Authors and year	Study Type	Asymptomatic aneurysms	Management		Radiological Outcomes		Clinical Outcomes	
			W&S	Treat	Untreat	Treat	New Symptoms	Stable
Kupersmit et al., 1992	R	12	12	0	Growth (1)	-	1	11
Stiebel-Kalish et al.. 2005	R	36	36	0	Stable (36)	-	5	31
Van Rooij et al., 2012	R	21	0	21	-	Occlusion (20)	1	20
Rosi Junior et al,. 2014	R	61	61	0	Thrombosis (6) Bone erosion (6)	-	13	48
Guan et al., 2016	R	51	0	51	-	Occlusion (45)	0	51
Miyachi et al., 2017	R	15	0	15	-	-	1	14
Vercelli et al., 2018	R	91	91	0	Growth (4) Stable (87)	-	4	87
Kumar et al., 2019	R	40	0	40	-	Occlusion (40)	N/A	N/A
Lee et al. 2021	R	6	0	6	-	Occlusion (6)	0	6
Fehrenbach et al., 2022	R	23	17	6	N/A	Occlusion (1)	0	6
Leivo et al., 2023	R	201	201	0	N/A	N/A	0	201

### Complication rates

As summarized in Fig. 4, complications occurred in both management arms. New or worsened visual symptoms were the most common adverse event, affecting 8.1% of conservatively managed patients and 9.4% of those treated actively. Ischemic events, including transient ischemic attacks and cerebral infarcts, occurred exclusively in the treated group at a rate of 2.9%. No cases of bleeding were reported in either cohort. Other complications in the treated group were rare and included cranial nerve palsy (1 patient) and fistula formation (1 patient).

### Risk of bias assessment

**Fig. 3:**
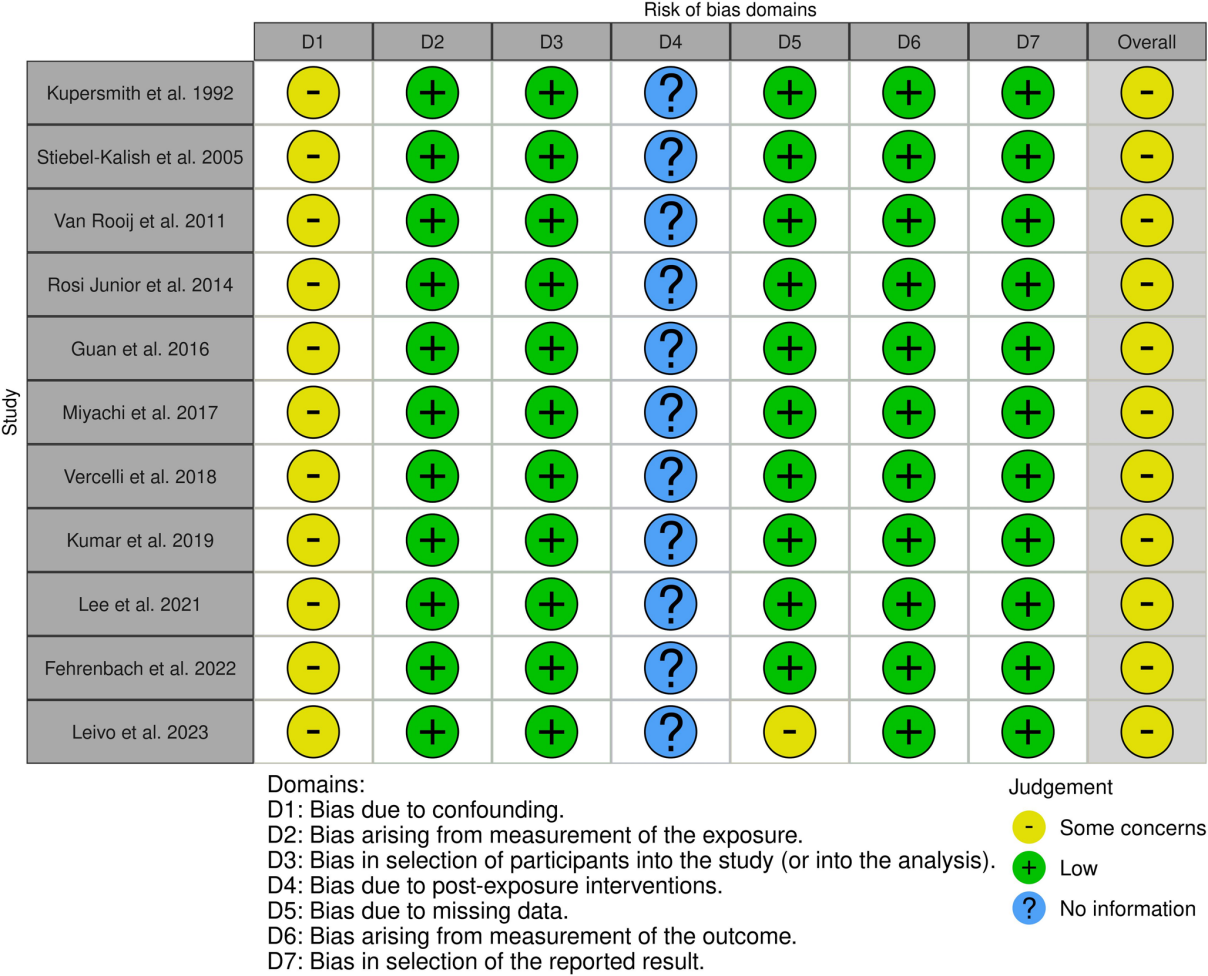
ROBINS-E (Risk Of Bias In Non-randomized Studies - of Exposure)

**Fig. 4:**
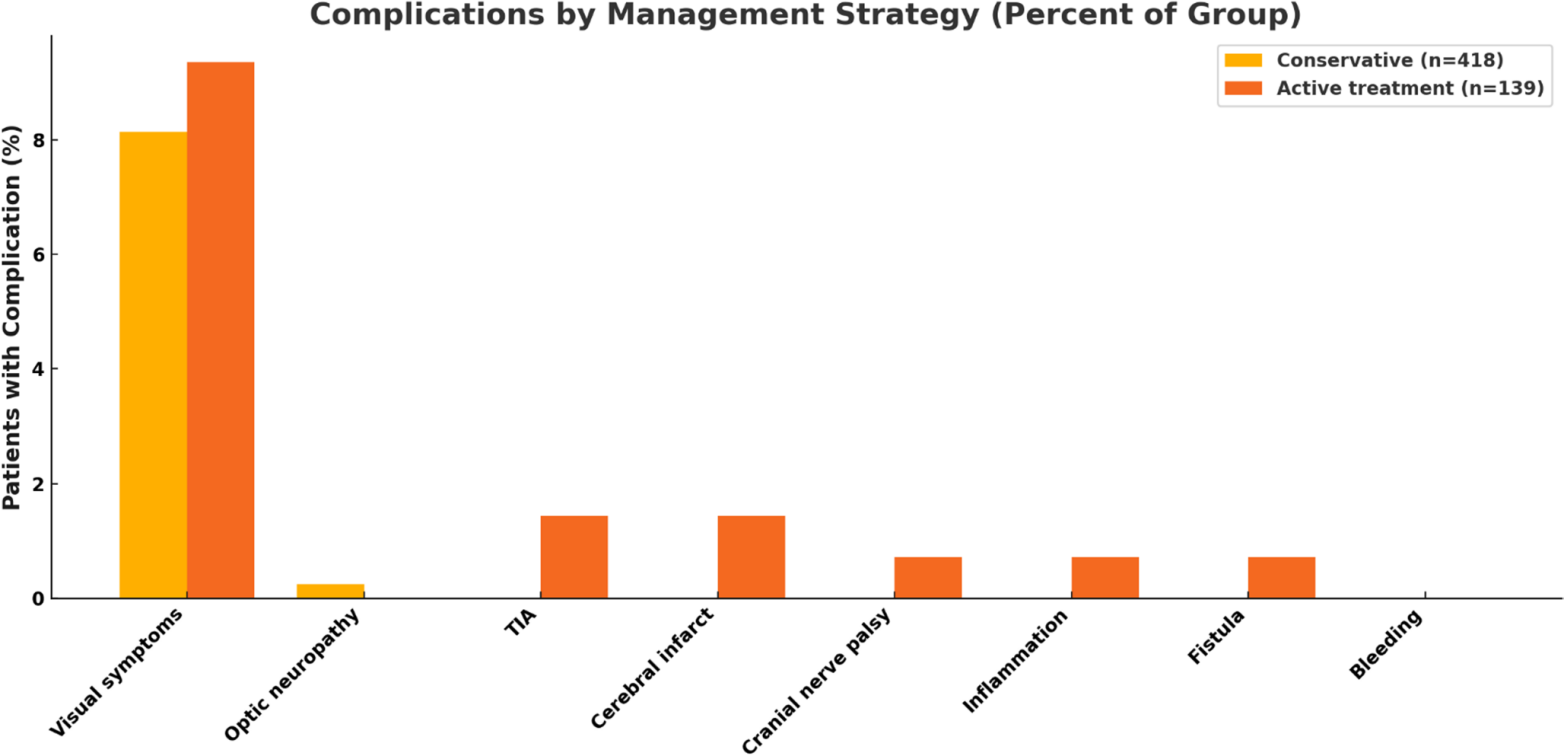
Complications by Management Group (Percent of Group)

The ROBINS-E assessment of the 11 included studies revealed an overall risk of bias of “some concerns” for all articles. This was consistently driven by a high risk of bias due to confounding, as the observational nature of the studies precluded control over the factors influencing the choice between conservative and active management. Other domains generally showed a low risk of bias (Fig. 3 and 4).

## Discussion

This systematic review, leveraging several large retrospective series with long-term follow-up, provides substantial data to address the clinical dilemma of managing asymptomatic CCAs.

### Natural history and the role of aneurysm size

The principal finding is that the natural history of asymptomatic CCAs is overwhelmingly benign, but it is dictated almost entirely by aneurysm size.

For small aneurysms, the risk of growth or symptom development is very low. The large cohort from Leivo et al. ^12^ provides strong evidence that a strategy of conservative observation for asymptomatic patients is safe over many years, with a near-zero risk of rupture or mortality related to the aneurysm, confirming classic teaching [10]. This is also consistent with the landmark International Study of Unruptured Intracranial Aneurysms (ISUIA), reporting a 0% five-year rupture rate for CCAs under 25 mm [18]. While the ISUIA findings offer this crucial context, the study was not included in our meta-analysis because its data for the CCAA subgroup were not presented in a way that allowed for a clear separation of outcomes based on conservative versus active treatment strategies.

However, the data clearly show that this benign course does not apply universally to larger lesions. As demonstrated convincingly by Vercelli et al.^17^, aneurysms exceeding 12 mm enter a different risk category, with a nearly 20% annual chance of growth.

Our analysis confirms the benign nature of these lesions. For the 418 patients managed conservatively, the clinical course was largely stable, with no reported ruptures leading to subarachnoid hemorrhage. We did, however, find that 34 patients (8.1%) developed new neuro-ophthalmic symptoms during follow-up. Analysis of the radiological data revealed that while the vast majority of aneurysms remained stable (91.5%), a small number underwent spontaneous thrombosis (3.0%). This phenomenon has been previously described and can correlate with episodes of severe pain followed by symptom improvement [10].

Another study focusing specifically on growth risk found that large or giant aneurysms (≥ 12 mm) had a 19.2% annual risk of growth, a figure dramatically higher than the 1.2% risk for very small aneurysms (< 4 mm) [17].

Our findings are strongly supported and contextualized by the recent large-scale meta-analysis by Shahbandi et al.^5^. Their work, focused exclusively on conservatively managed cohorts, reported an annual incidence rate of aneurysm growth of 2.91 per 100 person-years and a rate of new or worsened non-cerebrovascular symptoms of 3.41 per 100 person-years. Crucially, our review extends beyond natural history to provide pooled data on the outcomes of active intervention, a cohort not analyzed by Shahbandi et al. This dual analysis is the primary contribution of our work. By presenting the 85.7% pooled occlusion rate alongside the documented procedural risks (e.g., 2.9% ischemic events), our review provides clinicians with a balanced, evidence-based view of both sides of the management dilemma.

Additionally, it is important to acknowledge the potential of CCAs to evolve into a carotid–cavernous fistula (CCF). In our pooled cohort, no cases of spontaneous CCF occurred during conservative follow-up, whereas one treated patient (0.7%) developed a procedure-related fistula. Aneurysmal rupture into the cavernous sinus can give rise to a direct CCF, creating a high-flow shunt between the internal carotid artery and the cavernous sinus. This event leads to the sudden onset of ocular congestion, pulsatile exophthalmos, and cranial nerve palsy, and typically requires urgent endovascular treatment.

In contrast, indirect (dural) CCFs are low-flow connections between small dural branches of the internal or external carotid arteries and the cavernous sinus. They are often spontaneous, idiopathic, and may resolve spontaneously or respond to conservative therapy [19].

Recognizing this distinction highlights the continuum between aneurysmal pathology and arteriovenous fistulization within the cavernous sinus compartment [19].

These results allow for a more direct discussion with patients regarding the conservative strategy versus proactive treatment, a question not fully addressed by natural history data alone. Therefore, our manuscript serves as a complementary and necessary resource, offering a pragmatic summary of the expected outcomes for each major management pathway for asymptomatic CCAs.

### Efficacy and risks of active intervention

The management of a growing but still asymptomatic CCA remains controversial.

Our meta-analysis demonstrates that active treatment achieves a high rate of complete aneurysm occlusion (85.7%) with a low risk of serious complications, supporting the safety of this approach in well-selected asymptomatic patients [8]. The choice of intervention has evolved significantly, with a clear shift towards endovascular techniques. While a 2015 meta-analysis comparing endovascular coiling versus parent artery occlusion (PAO) found that PAO had higher occlusion rates without a significant difference in morbidity [20], this comparison predates the widespread adoption and technological refinement of flow diversion. More recent literature suggests that flow diversion is a highly effective and low-risk treatment, with one study of symptomatic patients reporting 88.4% complete or near-complete occlusion and 84.2% improvement in visual symptoms [3].

Moreover, while our review focuses on asymptomatic cases, the robust literature on treating symptomatic compressive aneurysms with flow diverters provides critical insight into the technology’s efficacy and risks. A recent large meta-analysis found that flow diversion led to improvement of neuro-ophthalmological symptoms in approximately 75% of patients. However, this efficacy is balanced by significant risks, with the same study reporting treatment-related morbidity in 5% and mortality in 3.9% of patients [21]. Furthermore, the timing of intervention in this cases appears paramount. Recent studies have demonstrated that a shorter duration of pre-treatment symptoms is a key predictor of better outcomes, with treatment initiated within one month of symptom onset increasing the likelihood of improvement more than tenfold. The mechanism for this improvement is strongly correlated with aneurysm shrinkage within the first three months post-procedure. This evidence from symptomatic cohorts suggests that while flow diversion is a powerful tool, its risks are not negligible, and its benefits are maximized when applied promptly after symptoms appear. This may support a strategy of careful observation for truly asymptomatic patients, with a low threshold for rapid intervention should they become symptomatic [21–23].

### Limitations of the review

The primary limitation is the retrospective nature of all included studies, which introduces significant selection bias and operational differences in the definition of “asymptomatic” across cohorts. For this reason, we avoided direct statistical comparisons between the two management arms. Additionally, significant heterogeneity exists among studies in terms of patient selection and follow-up duration (ranging from 6 months to 46 years) [12], which complicates the comparability of long-term outcomes. The wide variation in follow-up duration may have affected the pooled estimates. Shorter follow-up periods are less likely to capture late aneurysm growth or delayed symptom onset, while longer follow-up in some studies may overrepresent stable cases and give an impression of greater long-term safety.The inability to disaggregate complication data by specific treatment modality in most source studies prevents a more granular analysis of procedural risks, in particular precluded direct statistical comparisons between the two management arms. Funnel plots for assessing publication bias were not feasible due to the limited number and heterogeneity of included studies. Furthermore, clinical outcomes such as diplopia and trigeminal pain are inherently subjective and were not evaluated using standardized criteria across studies. This lack of uniform outcome assessment calls for cautious interpretation of the pooled results.

## Conclusions

This systematic review provides substantial evidence to guide the management of asymptomatic CCAs, confirming a clear dichotomy in natural history based on aneurysm size.

For small (< 12 mm) asymptomatic CCAs, the natural history is exceptionally benign, with a very low risk of growth or symptom development. Conservative management with serial imaging is a safe and appropriate strategy.

For large (≥ 12 mm) or growing asymptomatic CCAs, there is a significant and quantifiable annual risk of growth associated with the onset of neurological symptoms. In these patients, prophylactic endovascular treatment should be considered, balancing the risks of progression against the procedural risks .

This risk-stratified approach, grounded in the best available evidence, moves beyond prior uncertainty and allows for more nuanced, individualized patient care. Future prospective studies with standardized methodologies are needed to further refine these management strategies .

## Data Availability

rough data are available upon request.

## Abbreviations

CCAs: Carotid cavernous aneurysms
ROBINS-E: Risk Of Bias In Non-randomized Studies - of Exposure
ISUIA: Unruptured Intracranial Aneurysms
